# Intelligent Sensing in Dynamic Environments Using Markov Decision Process

**DOI:** 10.3390/s110101229

**Published:** 2011-01-20

**Authors:** Thrishantha Nanayakkara, Malka N. Halgamuge, Prasanna Sridhar, Asad M. Madni

**Affiliations:** 1 Division of Engineering, King’s College, University of London, London, UK; 2 Melbourne School of Engineering, The University of Melbourne, Melbourne, VIC, Australia; E-Mail: malka.nisha@unimelb.edu.au; 3 Microsoft Corp., Redmond, WA, USA; E-Mail: prassi hs@yahoo.com; 4 Crocker Capital, San Francisco, CA, USA; E-Mail: ammadni@yahoo.com

**Keywords:** sensor network, Markov decision process, sensing, reward shaping

## Abstract

In a network of low-powered wireless sensors, it is essential to capture as many environmental events as possible while still preserving the battery life of the sensor node. This paper focuses on a real-time learning algorithm to extend the lifetime of a sensor node to sense and transmit environmental events. A common method that is generally adopted in ad-hoc sensor networks is to periodically put the sensor nodes to sleep. The purpose of the learning algorithm is to couple the sensor’s sleeping behavior to the natural statistics of the environment hence that it can be in optimal harmony with changes in the environment, the sensors can sleep when steady environment and stay awake when turbulent environment. This paper presents theoretical and experimental validation of a reward based learning algorithm that can be implemented on an embedded sensor. The key contribution of the proposed approach is the design and implementation of a reward function that satisfies a trade-off between the above two mutually contradicting objectives, and a linear critic function to approximate the discounted sum of future rewards in order to perform policy learning.

## 1. Introduction

A sensor network is a network of spatially distributed sensing devices used to monitor environmental conditions (such as, temperature, sound, vibration, pressure, *etc*.) over a vast geographic area. Due to the tiny and inexpensive nature of construction, and due to low-power, low memory and low computational resources, these sensing devices are produced and deployed in large numbers. Various research problems of sensor networks such as data aggregation or fusion [1,2], packet size optimization [3], cluster formation [4,5], target localization [6], battery management [7], network protocols [8–10] are discussed in the literature with respect to crucial energy limitations. Efficient battery management for sensor lifetime [7] and guidelines for efficient and reliable sensor network design is investigated in [11]. A sensor node in an ad-hoc network can contribute to the network as long as its on-board power survives. It is known that almost 60% of the power is wasted on staying awake to communicate with other nodes [7]. However, if there are environment events that occur when the sensor nodes are not awake, the sensor does not transmit any new information. Let us consider a wireless sensor network of *N* sensor nodes. If each sensor is in the ON state, energy consumption is given by $E=\sum_{i=1}^{N}T_{i}P_{i}$ where *T_i_* is the period of time and *P_i_* is the power. In a general ON*−*OFF cycle, the sensor will do the following:

1. Sleep *−* shut down the power to all circuits except the clock
2. Wake up *−* supply power to all circuits
3. Read sensory information through the relevant ports. Ex. Analog to Digital conversion (ADC) port
4. Communicate the data to a remote node
5. Go back to sleep.

Environmental events that are random in nature (such as a tremor), require continuous sensing. If a sensor node deterministically sleeps, then it could miss critical events during the sleep period. However, time spent awake by the sensor, does not guarantee a random event in the environment—which drastically drains the on-board power of the sensor. Therefore, compromising the two conflicting objectives of staying awake to increase the probability of capturing critical information and conserving limited on-board energy to prolong the service time is generally a challenging task. Assume a sensor node is required to choose an optimum strategy of sleeping to preserve on-board power as long as possible while maximizing the chances of capturing important changes in the environment. Since the desired behavior is not known, the sensor can learn based on rewards and punishments in a reinforcement based learning paradigm [12]. First we design a reward function to assess the suitability of the sleep/awake cycles of the sensor. The paper is organized as follows. In Section 2, we predict future rewards using Markov Decision Process and in Section 3, we describe enhanced critic function. In Section 4, we obtain error estimation of offline critic function and outlines numerical and experimental results where we demonstrate the benefit of our new reward function and Section 5 concludes the paper.

## 2. Prediction of Future Rewards using Markov Decision Process

Markov decision process (MDP) is a stochastic process and is defined by the conditional probabilities [13]. This presents a mathematical outline for modeling decision-making where results are partly random and partly under the control of a decision maker. Broad ranges of optimization problems are solved using MDPs via dynamic programming and reinforcement learning [14]. In our work we use a reinforcement learning method.

When quantified supervisory feedback is unavailable, reward based or reinforcement based machine learning technique has proven to be an effective method [12,15–17]. The method is inspired by mathematical properties of the relationship between firing patterns of Dophamine neurons in the Basal Gangia and the timing of actions and external rewards or punishments to learn behaviors through ones own interaction with the environment [18–20]. However, it has been a long drawn out quest to find methods to design mathematical reward functions that are best suited to learn a given behavior [21–23]. In many cases, machines have to learn behaviors that satisfy an array of objectives sometimes with conflicting interests [23]. We encounter the problem in this application, where a sensor node is required to meet two conflicting objectives—one is to gather as much as information from the environment as possible, and the other is to survive as long as possible with limited on-board energy. Consider a sensor node to capture ambient temperature changes. We can take the ratio between the battery voltage at a given time and that at full charge given by *W_k_/W_max_* as a measure of the risk of losing functionality due to battery drainage. Therefore, lower values of this ratio should reduce the reward level assigned for capturing new information. The reward assigned for capturing new information should increase with the increasing difference of measured temperature Δ*T* = |*T*_*i*+1_ − *T_i_*| between two consecutive waking up times (*i, i* + 1). However, the reward should also increase if new information is captured by staying awake for bellow average lengths of time. Therefore the sleeping behavior can be quantified by the ratio between the current sleep time and the average sleep time defined by the user given by *Sl_t_/Sl_ave_*. Combining the above requirements of on-board energy conservation and gathering new information, the reward function *r*(*k*) can be formulated as shown in Equation (1).

(1) $$r(k)=\left(\frac{W(k)}{W_{\max}}\right){\left(\frac{\mathrm{Sl}(k)}{{\mathrm{Sl}}_{\mathrm{ave}}}\right)}^{\xi \left|T_{k}-T_{k-1}\right|}$$

where *W* (*k*) and *W_max_* are battery voltage at time *k* and maximum battery level, *Sl*(*k*) and *Sl_ave_* are sensor sleep time at time k and average sleep time and *ξ* is a scalar parameter. Here we assume a battery where the open circuit voltage reflects the useful energy level. However in any other case, a measurable variable that represents the useful energy level can be used here. According to Equation (1), if the on-board energy level drops (drop of *W* (*k*)/*W_max_*), the only way to obtain higher rewards is to go for an above average sleeping strategy so that it still captures significant temperature changes (Δ*T* = |*T_k_* − *T*_*k*−1_|) in the environment between two consecutive wake-up instances. However, this reward function cannot achieve a long term optimality of the sleeping behavior of the sensor. Therefore, we should design a critic function that estimates the total future rewards generated by the above reward function for an agent following a particular policy. The total expected future rewards *V̂*(*t*) given by

(2) $$\begin{array}{ll} \hat{V}(t) & =\sum_{k=0}^{\infty }\gamma^{k}r(t+k)\;\text{which can be expanded to give}\\ \hat{V}(t) & =r(t)+\sum_{k=1}^{\infty }\gamma^{k}r(t+k),\\ & =r(t)+\gamma \sum_{k=0}^{\infty }\gamma^{k}r(t+k),\\ & =r(t)+\gamma \hat{V}(t)(t+k) \end{array}$$

**Figure 1.**
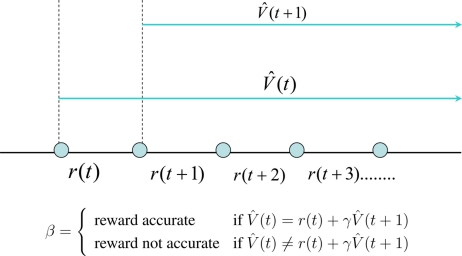
Temporal difference based learning to predict.

Equation (2) holds true only if the prediction function *V̂* (*t*) is accurate. If not *V̂* (*t*) ≠ *r*(*t*)+*γV̂* (*t*+1). This prediction error as a *temporal difference* (TD) is given by Δ = (*r*(*t*) + *γV̂* (*t* + 1)) − *V̂*(*t*) as shown in Figure 1.

An optimum value function can be learnt by updating the internal parameters of the function to minimize the temporal difference error Δ. Since Δ is a known quantity, learning the value functions are strictly a supervised learning mechanism. However, it learns how a qualitative reward function behaves with states and actions. It should be noted that the ability to predict is an essential ingredient of intelligence because it allows us to explore for better behavioral policies without waiting for the final outcome. When a human baby learns to do various things like walking, jumping, cycling, *etc*., the parents often use their experience to predict the outcome of their actions. This helps the child to learn till he builds up his own internal models of the world to predict the outcomes of his actions. This internal or external predictor is commonly known as a critic. The job of the critic is to estimate the value function *V̂* (*t*). Now let us discuss how a critic can help an agent to learn an optimum policy.

**Figure 2.**
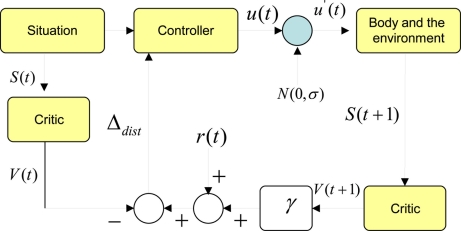
Actor−critic based learning: using the ability to predict to improve the behaviors (control policy). Here is a sleeping policy of the sensor node.

Assume a critic function has learnt to predict total expected future rewards at any given state using a temporal difference based learning scheme. Figure 2 shows how a critic can be used to evolve an optimum controller or a policy. In this case the action space is a continuous one. We start with a particular situation. In our terms a situation can be simplified to a Markov state. Given this state, the trained critic predicts the total expected future rewards known as the value of the state *V* (*t*). On the other hand, the policy can take an action. For the time being, we do not know if this action *u*(*t*) is the best one to take given the current state. Therefore, we add a normal distributed exploratory disturbance *N*(0, *σ*) the action, where *σ* is the width of the distribution. Since this distribution is centered at the origin, the disturbance can increase or decrease the value of the action. If it is a robot, the action is the torque/force commands to the actuators. Once this modified action is fed to the actuators, the robot enacts a behavior that interacts with the environment. The resulting new situation can be characterized by a new Markov state. If we feed this new state to the same critic, it should give us the expected total future rewards from the next point of time denoted by *V* (*t* + 1). Since we obtain a real reward value at this point, we can compute a temporal difference of prediction induced by the disturbance. We denote this by Δ*_dist_* = (*r*(*t*) + *γ V̂*(*t* + 1)) *− V̂*(*t*). It should be noted that we assume that the critic is well trained so that this temporal difference of prediction would be zero if the disturbance was not given.

**Figure 3.**
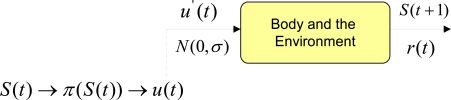
How the temporal difference can be used to improve the policy. Here *u*(*t*) = *π*(*s*(*t*)).

If Δ*_dist_* is a positive value, that means the modified action and the new state has been better than what was expected when the critic predicted *V* (*t*). This will be true if and only if (*r*(*t*) + *γV* (*t* + 1)) > *V̂* (*t*). This implies that the controller or the policy should change its internal functionality to be one that would produce an action closer to the modified action given the state *S*(*t*). Here state *S*(*t*) is the average change of temperature detected by the sensor during an awake cycle. If the modified action was detrimental in the eyes of the reward function, this would be reflected by a negative Δ*_dist_*. In that case, the policy should try to move away from generating an action in the direction of the modified action given the state *S*(*t*). This can be seen in Figure 2.

$$\begin{array}{l} \Delta_{\mathrm{dist}}>0\;S(t)\to \pi^{\prime }(s(t))\to \pi (s(t))+\eta (u^{\prime }(t))-\pi (s(t))\\ \Delta_{\mathrm{dist}}>0\;S(t)\to \pi^{\prime }(s(t))\to \pi (s(t))-\eta (u^{\prime }(t))-\pi (s(t)) \end{array}$$

where *η* is a scaler. According to Figure 3, the policy updating rule can be generalized as given by *π′* = *π* + *η*Δ*_dist_*(*u′*(*t*) − *u*(*t*)).

### 

#### Simplification−1

Let us have a closer look at the actual function that the critic is trying to estimate. Ideally the total expected future rewards is given by

(3) $$V(k)=\sum_{m=0}^{\infty }\gamma^{m}r(k+m),\;\gamma =0.6.$$

The choice of a value for *γ* depends on the user’s prior expectation of the uncertainty of the environment. Lower the value, the shorter the effective span of future rewards that will be accounted for in the computation of the total expected future rewards *V* (*k*). Here we choose *γ* = 0.6. We observe that *γ*^1^ = 0.6*, γ*^2^ = 3.6*, γ*^3^ = 0.216*, γ*^4^ = 0.126*, γ*^5^ = 0.077*, γ*^6^ = 0.046. This implies that any future reward value beyond the sixth time step will be discounted by less than 4%. Therefore, we can reasonably assume that we take only those future reward values upto the sixth time step. This reduces the complexity of estimating the critic function. However, this assumption depends on the uncertainty of the environment which is reflected in the value of the discounting factor. If discounting factor *γ* = 0, then the learning agent is extremely uncertain about the future so that it does not even dare to predict what will happen in the next time step. If the environment is quite predictable, the value of *γ* approaches 1.

## 3. Enhanced Critic Function

**Figure 4.**
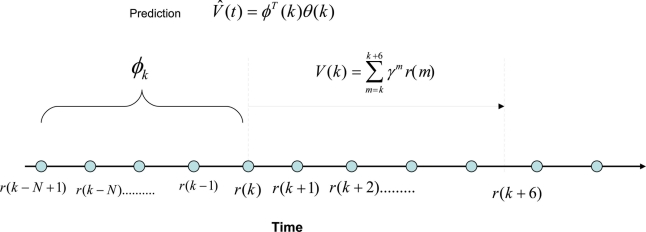
The structure of the polynomial critic function.

Here we investigate the possibility of developing an autoregressive function to estimate *V* (*k*) given by, where *V̂*(*k*) = *ϕ^T^* (*k*)*θ*(*k*), where *ϕ*(*k*) = [*r*(*k*) *r*(*k* − 1) ⋯ *r*(*k − N* + 1)]*^T^* is a vector of past rewards, and *θ*(*k*) is a vector of scalar parameters of the same size as *ϕ*(*k*). Due to the limitation of computational resources available for the embedded sensor to estimate the critic function, we choose to train the critic off-line. In order to do that, we can take a series of training data in different environmental conditions. Figure 4 shows how this prediction mechanism works. The polynomial function predicts the total expected discounted sum of future rewards at time *t* = *k* using the past rewards. Then we take six data points from *t* = *k* to calculate the actual value function for *γ* = 0.6. It should be kept in mind that this can be done because we are training the critic off-line using batches of data obtained from experiments. Therefore the prediction error is given by *ɛ* = *V* (*k*) *− V̂* (*k*). Here, we assume that the nonlinear dynamic behavior of the sensor and the environment can be approximated by a linear combination of the non-linear static reward functions. The importance of this assumption is that the simplified linear dynamic regression function can be easily implemented on an embedded sensor with limited processing and memory capacity because it only requires remembering a short array of past reward values and a similar number of polynomial parameters.

## 4. Error Estimation of Offline Critic Function

The following recursive least squares algorithm is used to optimize the polynomial parameter vector *θ*(*k*).

(4) $$\begin{array}{l} \theta (k)=\theta (k-1)+P(k)\phi (k-1)\left[V(k)-\phi {\left(k-1\right)}^{T}\theta (k-1)\right]\\ P(k)=P(k-1)-P(k-1)\phi (k-1){\left[1+\phi {\left(k-1\right)}^{T}P(k-1)\phi (k-1)\right]}^{-1}\phi {\left(k-1\right)}^{T}P(k-1)\\ P(k)\in {ℛ}^{N\times N} \end{array}$$

The above least squares estimation algorithm was run for different orders of the polynomial from 2 to 7. We observe that the total estimation error across all data sets behaved for different orders of the polynomial. Based on the simulation results and experimentations, it was noted that order 4 polynomial is best suited with a minimum average estimation error $ɛ=\sum_{k=1}^{T}||(V(k)-\hat{V}(k))||$, where *T* is the total time span.

### 4.1. Experimental Validation of the Critic

**Figure 5.**
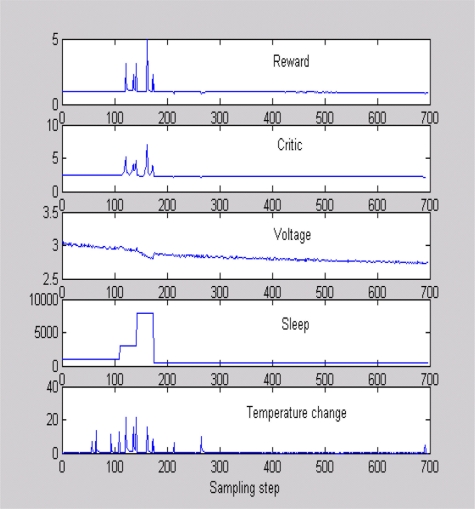
Evaluations of reward and critic.

**Figure 6.**
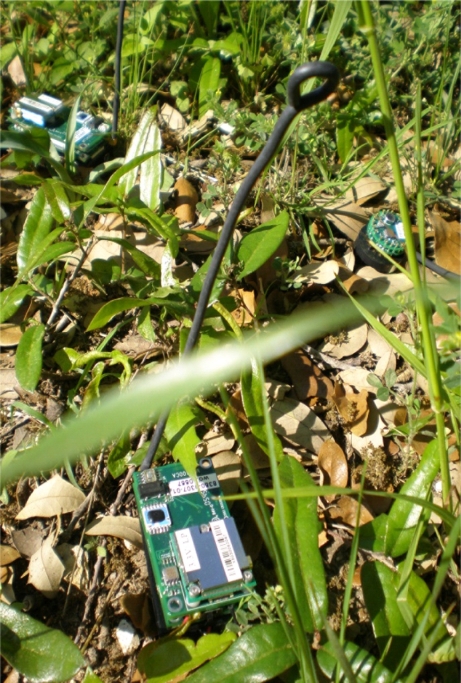
Implementation of reinforcement learning on sensors in an outdoor environment, by using MTS400 CA embedded board with external antenna.

Figure 5 shows a typical scenario used to train the critic, where sleep time and temperature were changed and data were obtained when the battery voltage was dropping as in [24]. Figure 5 is helpful in evaluating the quality of our reward function. It can be seen that the reward increases when the sensor detects a high temperature difference with a high sleep time. This means that the sensor is awake to capture an important event, thanks to an adaptive sleeping strategy. Similarly, the reward is low with temperature changes when the battery voltage is low. Once the critic is trained, we program the sensor to keep the polynomial equation to estimate the total expected future rewards for a chosen sleeping strategy. In the real-world implementation, the sensor will calculate the critic value for several different sleep times after every four sampling time steps. It will implement the sleep time that gives the best critic output. All experiments were performed on a *MT S*400*CA* embedded board with 128 kb memory and 16 MHz processing speed show in Figure 6.

### 4.2. Extension to Clusters of Sensor Nodes with a Simplified Real-Time Markov Decision Making Process

With critic loaded on the sensor node, a 10 times reduction in the number of packets transmitted was achieved with a very few misses in registering the events. The events registered (*i.e.*, temperature reading captured) with and without critic is observed. The number of transmitted packets with and without critic running on sensor node is as shown in Table 1. We examine, there are only few temperature differences missed by using adaptive sleep time. Whenever there is a change in temperature registered, the sleep time is automatically decreased so as to capture the change with a finer resolution. In a general case, this can be thought of as sensor adaptively waking up based on the environmental changes in order to localize the events.

### 4.3. Real-Time Decision Making in Clusters of Sensor Nodes

We further simplify the Markov decision making process and extend the real-time learning algorithm to a cluster of sensors that can choose the most optimum one out of several possible sleep strategies.

#### Initialize variables:

*V_i_* is the discounted sum of past rewards for the *i^th^* sleeping strategy, *γ* is the discounting factor, *R_i_* is the reward assigned to the *i^th^* sleeping strategy *S_i_*.

**Step-1:** Use the measurements obtained during an awake cycle to compute the reward for the chosen sleep strategy *S_i_*. The reward is given by *R* = *I*(*E/E_max_*), where *I* is the total information gathered during the awake period, *E* is the remaining on-board energy, and *E_max_* is the energy at full charge. The gathered information *I* is the mean unsigned temperature change detected by the sensor. Therefore, given an energy level a sensor receives higher reward if it matches awake time to suit the statistics of the environment. We achieve a compromise between the two conflicting objectives of preserving on-board energy by sleeping on one hand and to gather as much information as possible by staying awake on the other, by taking the product of the two quantities *I* and (*E/E_max_*).

**Step-2:** Compute the discounted sum of rewards for all possible sleeping strategies given by *V_i_* = *V_i_γ* + *R_i_*.

**Step-3:** Share locally *V_i_* computed values among all sensor nodes in a local cluster at any given time. Use Roulette wheel selection strategy to choose a sleeping strategy at the node level.

**Step-4:** Use a 10% probability to make a deviation from the chosen sleeping strategy to the next best strategy at a sensor node level. Therefore, different nodes may make different deviations from the optimal solution for the cluster.

**Step-5:** Go to Step-1.

Our purpose here is to achieve the reward function defined by the following multiple objective unconstrained optimization problem:

$$\underset{k\in K}{\text{max}}(1-\alpha )R(k)-\alpha ɛ(k),$$

where *K* is define the set of all possible time, $K=\left\{k_{i}|0<i\leq \frac{\partial E}{{\partial P}_{i}}\right\}$ and *α* ∈ [0, 1] the weight factor designates the relative significance of the two objectives *R*(*k*) and *ɛ*(*k*).

### 4.4. Simulation Results for a Cluster of Three Sensor Nodes

We performed simulations for three sensor nodes that can adaptively choose from three different strategies of sleeping duty cycles given by [10%, 50%, 100%]. In order to test the ability of the learning strategy to adapt itself under changing environmental conditions, we use a sinusoidal ambient temperature profile that changes its frequency over time, given by *θ* = 2 + sin(2*πf*(*n*)*nT*), *f* = *n/KN*, *n* = 1*,* 2*,* 3*, ⋯, N*, where *θ* is the ambient temperature, *T* is the sampling interval, *n* is the sampling step, *N* is the total number of samples, and *K* = 10 is a constant. Apparently, the ambient temperature in this case starts at a high frequency sinusoidal pattern, and gradually progress to a low frequency sinusoidal pattern. Therefore, a learning sensor node should be able to change the duty cycle of sleeping to suit the rate of change of ambient temperature—low sleeping duty cycle at low frequencies and high sleeping duty cycle at high frequencies of ambient temperature. Each sensor node lost a fixed amount of energy in every awake sampling step so that if the nodes stay awake throughout the *N* sampling steps, they would lose all on-board energy.

**Figure 7.**
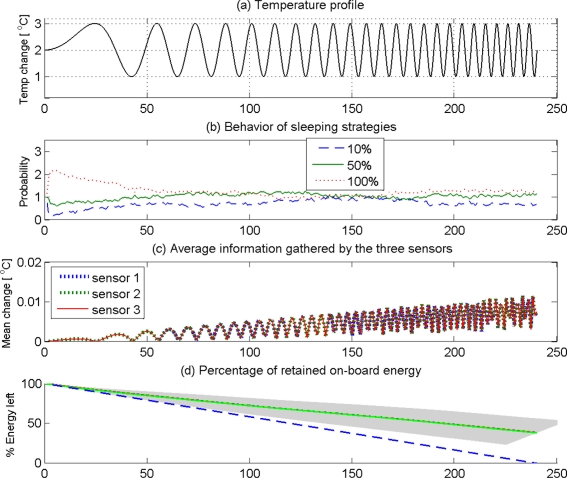
Adaptive behavior of a cluster of sensor nodes following a Markov decision process in a stochastic environment.

Figure 7 shows the simulation results for 100 trials of three sensor nodes staying a span of 250 seconds in each trial to detect as much changes in the ambient temperature as possible by learning to optimize sleeping behavior to retain as much on-board energy as possible. Figure 7(a) shows the ambient temperature profile that monotonically increases the frequency of sinusoidal fluctuations. Figure 7(b) shows the probability of using each sleep strategy (10%, 50%, and 100%) at any given time across the 100 trials. It should be noted in Figure 7(b) that 100% awake cycles have been chosen more often in the early stages (*<* 50 seconds). However, when the on-board energy drops (Figure 7(d)) the sensors have chosen to fall down to 50% and 10% awake cycles more often especially between 100*−*130 second interval. The blue line approaching zero in Figure 7(d) confirms that the sensors would have lost all on-board energy had they chosen 100% awake policy all the time. The other lines show how the sensors preserved on-board energy by optimal selection of sleeping strategies. However, it should be noted that the sensors have explored the viability of both 100% awake cycles in this period with more tendency to choose the former. This exploration has been more vigorous around 150 second where the sensors experienced a drop of on-board energy closer to (62 +/− 12)% of their original on-board energy. Towards the latter part (200 seconds) all sensors had preferred 10% awake policy that helped all sensors to lower the rate of loss of on-board energy (Figure 7(d)). What is important to note here is that sensors had not abandoned the explorative behavior even after taking the energy management under control. They have explored all policies after 210 seconds to capture more information in the rapidly changing environment. This is reflected in the fact that all three sensors have increased the average information captured as shown in Figure 7(c).

The above intelligent behavior emerged in a cluster of three sensors that used Markov decision process with a simple reward function that combined the two contradicting needs—to gather as much information as possible and to preserve as much on-board energy as possible—of a typical stand-alone sensor node. According to this approach, a cluster will have a general tendency to select the sleeping strategy that has accumulated the largest discounted sum of rewards. However, the explorative moves will enable the sensors to keep a track of the changing level of optimality of other strategies in a stochastic environment. Therefore, the Markov decision making strategy can automatically adapt to suit the statistics of the environment.

## 5. Conclusion

Real-time learning algorithm is developed to extend the lifetime of a sensor node to sense and transmit environmental events. The purpose of our new learning algorithm is to couple the sensor’s sleeping behavior to the natural statistics of the environment therefore it can be in optimal synchronization with changes in the environment by sleeping with steady environment and staying awake when turbulent environment. Theoretical and experimental validation of a reward based learning algorithm that can be implemented on an embedded sensor is presented. Our results show the inclusion of proposed learning algorithm is significantly more effective to preserve sensor on-board energy by optimal selection of sleeping strategies.

We have presented results for a network of three sensor nodes. However, the method can be extended to a generic *N* number of nodes. However, in that case, the algorithm will accurately reflect distributed phenomena in the environment if local clusters were formed to respond to local changes in the measured variables (temperature in this case). An optimal strategy to form such clusters should be based on the statistics of the environment, and is outside the scope of this paper. This paper discusses how such a local cluster would perform when the variation of the temperature increases its frequency over a 250 second time span. However, it can be readily extended to any time span because the in-built forgetting mechanism—through discounting past rewards—maintains the timeliness of the optimality of the cluster.

## Figures and Tables

**Table 1.** The comparison of performance with and without the critic based adaptive sleeping behavior.

Time	Number of Transmitted Packet	
	With Critic	Without Critic
0	0	0
50	100	600
100	120	1200
150	200	1850
200	260	2500
250	310	3100
300	400	3750
